# Crushing Muscles: A Case Study on Rhabdomyolysis, Renal Failure, and Compartment Syndrome Triggered by Pre-Workout Supplement Abuse

**DOI:** 10.7759/cureus.58775

**Published:** 2024-04-22

**Authors:** Faryal Altaf, Vedangkumar Bhatt, Sindhaghatta Venkatram, Gilda Diaz-fuentes

**Affiliations:** 1 Internal Medicine, BronxCare Health System, Bronx, USA; 2 Medicine and Surgery, BronxCare Health System, Bronx, USA; 3 Pulmonary and Critical Care Medicine, BronxCare Health System, Bronx, USA

**Keywords:** hematology, supplemental toxicity, myoglubineuria, hemodialysis, foot drop, renal failure, pre-workout, creatine, protein supplements, rhabdomyolysis

## Abstract

The use of steroids and protein-based dietary supplements for muscle enhancement is prevalent in contemporary society. While these products promise increased muscle mass and strength, they carry significant risks, including severe medical complications. The consumption of these supplements has been linked to adverse symptoms, including dehydration, gastrointestinal distress, dizziness, and alterations in heart rate and blood pressure, primarily due to ingredients like creatine, arginine, and caffeine. Following the proper dosage, ensuring adequate hydration, and consulting a healthcare provider to verify if the supplement's components could affect any pre-existing conditions is recommended. Indiscriminate use of these products, including taurine, can lead to serious side effects. We present a 36-year-old patient with severe rhabdomyolysis, life-threatening acid-base imbalance, renal and liver injury, and peripheral neuropathy associated with the use of performance-enhanced unregulated supplements and exercise. This case highlights the importance of recognizing and managing complications related to exercise-aid supplements, emphasizing early identification and management. Increasing social awareness and research on those products is highly needed to avoid supplement-associated complications and potential long-term disabilities.

## Introduction

In the fitness and bodybuilding communities, the utilization of steroids along with dietary supplements like protein powders is a common strategy aimed at augmenting muscle mass and enhancing strength [1]. The principal supplements are whey isolate protein, creatine, and vitamins [2]. Reported side effects of those supplements include exercise-induced acute renal failure, rhabdomyolysis, and gastrointestinal disturbances [3]. Increased dietary protein intake can potentially have a severe impact on renal function, causing glomerular hyperfiltration with elevated glomerular filtration rate (GFR) renal flow and increased proteinuria [3,4]. It can lead to an acceleration of chronic kidney disease (CKD). In this context, we discuss the case of a 36-year-old male who experienced severe complications following the consumption of a creatine monohydrate-rich pre-workout supplement. This case serves as a cautionary tale about the possible risks associated with using exercise supplements and underscores the importance of their proper identification and management.

## Case presentation

A 36-year-old male with hypertension and a history of renal stones was admitted to the ICU, presenting with progressive weakness and numbness in both lower extremities, accompanied by pain and tenderness. Notably, no saddle anesthesia or urinary or fecal incontinence was reported. The patient described the leg pain as "electric," originating from the left anterior hip and radiating to the foot, which was alleviated by rest and reduced by analgesics such as non-steroidal anti-inflammatory drugs (NSAIDs). Difficulty in walking was attributed to the pain. His lifestyle included occasional smoking and alcohol consumption, and he had been employed as a painter since relocating to New York a year ago. He maintained a regular exercise regimen and had been consuming a pre-workout supplement for the past two months, denying any excessive exercise or anabolic steroid use. Physical examination revealed normal vital signs, left foot drop, pitting edema, and diminished strength in the left lower extremity.

**Figure 1:**
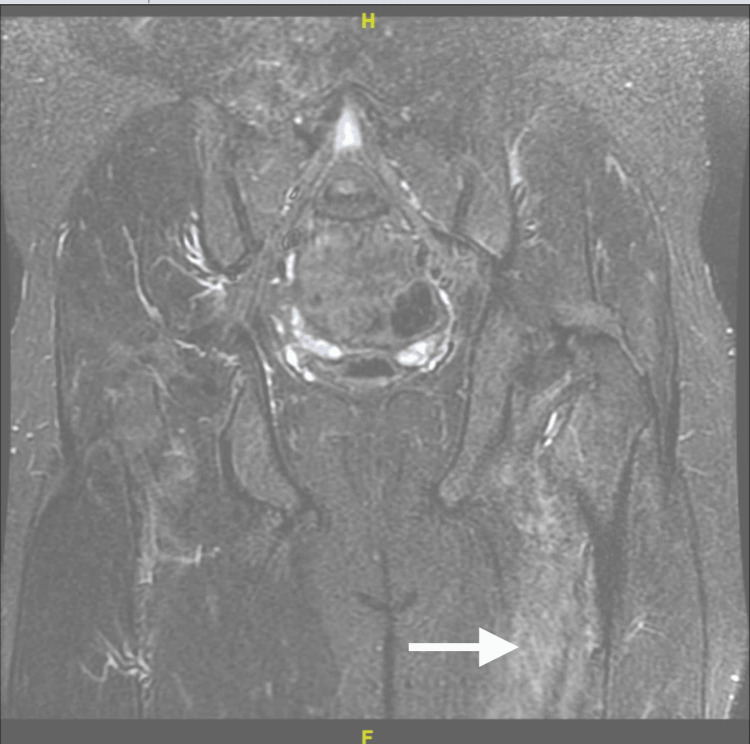
MRI of the lower extremity showing focal areas of intramuscular edema, including the lower lumbar spine posterior paraspinal musculature and bilateral pelvic musculature (left greater than right), concerning infectious myositis versus significant muscle strain.

The initial laboratory diagnostics revealed acute renal failure, elevated liver enzymes, increased serum creatine kinase (CK), and several acid-base imbalances, including pronounced hyperkalemia and anion gap metabolic acidosis, detailed in Table 1. The patient received therapy for hyperkalemia, IV crystalloid fluids, and high-dose dexamethasone for suspected foot drop. Computed tomography (CT) delineated anterolisthesis, lumbosacral spondylosis, moderate bilateral neural foraminal narrowing at the L5 nerve exits, and thoracic Scheuermann's disease. An exhaustive workup employing CT and magnetic resonance imaging (MRI) scans excluded spinal abscesses, cauda equina syndrome, transverse myelitis, stroke, and aortic dissection. MRI findings confirmed moderate foraminal narrowing and significant muscle strain, evidenced by bilateral edema in posterior paraspinal and pelvic muscles, depicted in Figure 1. Ultrasound examination for deep venous thrombosis in the lower extremities returned negative results.

**Table 1:** Summary of laboratory investigations BUN: blood urea nitrogen; INR: international normalised ratio; PT: prothrombin time; ANA: antinuclear antibody; SS-A AB: Sjögren's-syndrome-related antigen A autoantibodies; SS-B AB: Sjögren's-syndrome-related antigen B autoantibodies

Laboratory Test	Admission	After Hemodialysis	Discharge	Reference Values
Serum Sodium	131	140	135	135-145 mEq/L
Serum Potassium	7.3	4.8	4.9	3.5-5.0 mEq/L
Serum Creatinine	4.9	3.8	1.8	0.50-1.10 mg/dl
BUN	50.0	43	42	8.0-26.0 mg/dl
Calcium	8.3	9.6	8.8	8.5-10.5 mg/dl
Aspartate Transaminase, Serum	2168	560	37	9-48 unit/L
Alanine Aminotransferase, Serum	501	251	47	5-40 unit/L
Hemoglobin	19.4	11.7	10.1	12-16g/dl
White Blood Cell Count	17.6	11.8	7.2	4.8-10.8k/ul
Platelet Count	315	192	416	150-400 k/ul
PT	11.7	13.7	13.6	9.9-13.3 seconds
INR	1.01	1.18	1.17	0.85-1.14
Serum Creatine Kinase	84983	16472	495	20-200 units/L
Alkaline Phosphatase, serum	116	43	57	53-128 units/L
ANA	1:40			Negative
Vitamin B12	203			243-894 pg/ml
Thyroid Stimulating Hormone	0.28			0.40-4.50 Miu/l
T4	1.03			0.80-2 ng/dl
Complement 3	210			90-150mg/dl
Complement 4	52			16-47mg/dl
C- Reactive Protein	36			<5 mg/l
HIV 1/2/O Antibody	Non-reactive			
Myeloperoxidase	<1.0			<1 AI
Parathyroid Hormone	57			10-65 pg/ml
Proteinase-3 Antibody	57.50			<1 AI
SS-A AB	<1.0			<1 Negative AI
SS-B AB	<1.0			<1 Negative AI
Anti-Jo antibody	negative			<1 Negative AI
Anti-centromere Antibody	negative			<1 Negative AI

**Figure 2:**
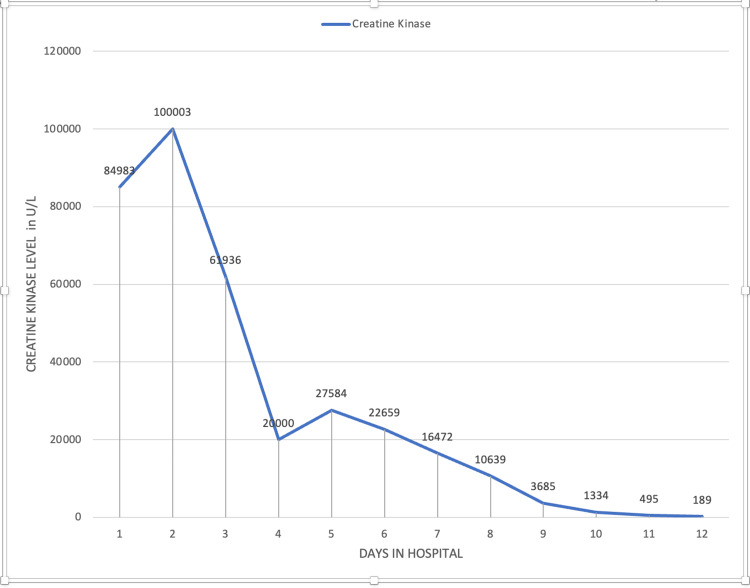
Trend of creatine kinase (CK) during the hospital stay. Hemodialysis was started on day 7.

**Figure 3:**
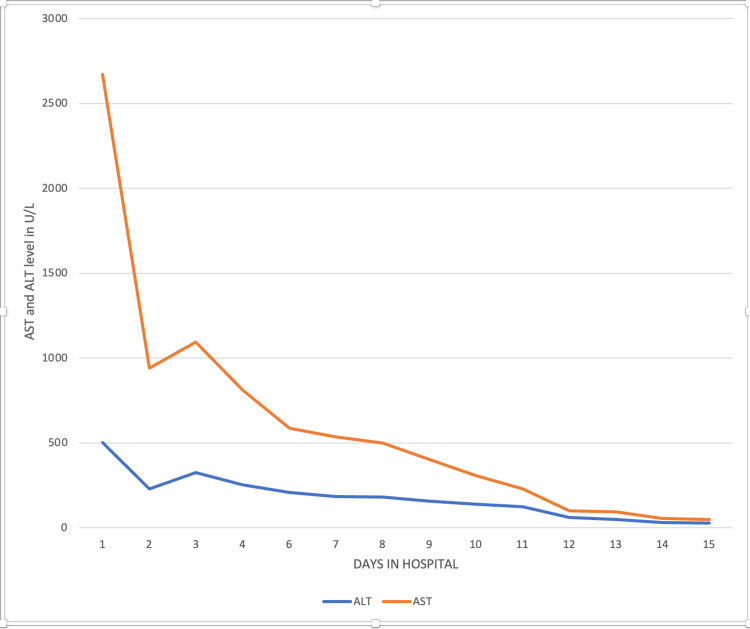
The trend of aspartate aminotransferase (AST) and alanine aminotransferase (ALT)

**Figure 4:**
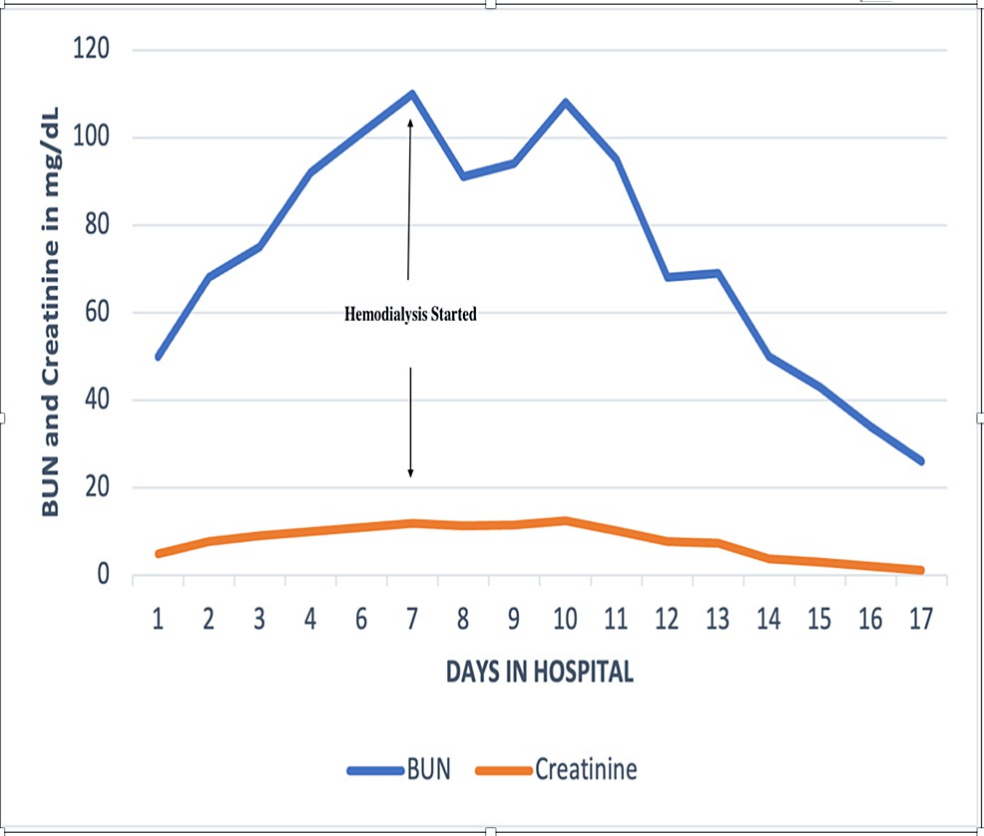
The trend of blood urea nitrogen (BUN) and creatinine during the hospital stay.

Despite intensive treatment of rhabdomyolysis, the patient experienced a decline in renal function, evidenced by ongoing hyperkalemia and reduced urine output. Compartment syndrome in the left leg was diagnosed, attributed to the rhabdomyolysis. Electromyography (EMG) of the lower limb revealed a subacute to chronic partial axonal injury of the left sciatic nerve (tibial/fibular) above the knee, with an estimated 50% axonal loss. This injury led to foot drop, directly linked to peroneal nerve damage resulting from the compartment syndrome. A joint decision by the patient and vascular services led to a conservative approach to managing compartment syndrome, avoiding fasciotomy. Hemodialysis was initiated on the fifth day of admission, comprising five sessions, which gradually lessened renal function and corrected laboratory abnormalities. The patient's condition progressively improved, with reduced leg swelling and amelioration of neurological symptoms and pain in the leg. He was eventually discharged to continue physical therapy and pain management at home, with no further need for hemodialysis. The trend of creatine kinase, aspartate aminotransferase (AST)/alanine transaminase (ALT), and blood urea nitrogen (BUN)/creatinine is shown in Figures 2-4.

## Discussion

The patient in the current case presented with severe rhabdomyolysis associated with severe electrolyte imbalance, compartment syndrome, and acute kidney injury requiring hemodialysis. Creatine/caffeine supplements and exercise likely contributed to and potentiated the development of rhabdomyolysis. Performance supplements have frequently been associated with negative impacts on overall health; however, these performance enhancers are extremely popular among health-conscious individuals despite the lack of current and reproducible outcome data [5]. In this case, the patient initially took an exercise aid rich in creatine monohydrate, a relatively common, widely available, and publicized pre-workout supplement. The first report raising/concerns regarding the safety of creatine was in 1997 when three college wrestlers cutting weight for qualifications used creatine as their supplement [6]. All three individuals died within two months, and the causes of death were listed as rhabdomyolysis [6,7]. Most supplements, including creatine, are not regulated in the United States and are classified as dietary supplements, making it crucial for physicians to inquire about the patient’s usage [8].

The etiology of rhabdomyolysis is generally classified into two categories: traumatic/physical causes and nontraumatic/nonphysical causes [9]. Some of the most common etiologies from the former group are motor vehicle accidents, prolonged immobilization, fractures of the lower extremities, burns, and electric shocks [10]. These patients generally have a poor prognosis compared to the non-traumatic group. Classic presentations of patients with traumatic etiologies will have increased levels of myoglobin, serum CK, and myoglobinuria. Nontraumatic causes of rhabdomyolysis include medications, recreational substances, and infections; colchicine, corticosteroids, anticholinergics, diuretics, statins, antipsychotics, antidepressants, and anesthesia medications have been associated with rhabdomyolysis [11]. Mycoplasma, legionella, HIV, toxic shock syndrome, and viral infections like influenza and COVID-19 are the common infectious cases related to rhabdomyolysis [12].

Rhabdomyolysis in nontraumatic instances, like our patient, usually occurs due to electrolyte and metabolic abnormalities and a mismatch between oxygen supply and demand; muscle injury is either a direct myocyte injury or an energy supply failure in the muscle cell [13]. The lack of ATP in nontraumatic causes and direct myocyte injury in traumatic causes leads to an influx of intracellular sodium and calcium, causing cell swelling and increased activation of actin-myosin cross-linkage, myofibrillar contraction, and eventual depletion of ATP. This is associated with reperfusion with excessive intracellular calcium, causing cell membrane disruption leading to further myolysis and necrosis of muscle fibers. A sequence of clinical disorders occurs such as acute kidney injury, following the release of muscle breakdown products such as myoglobin, CK, potassium, and uric acid [13]. Cellular injury in rhabdomyolysis causes a discharge of myocyte contents, resulting in renal damage due to renal tubular obstruction, renal vasoconstriction, and oxidant, causing direct tubular dysfunction [14]. Five to seven percent of acute renal failure in the United States is caused by rhabdomyolysis, which is one of the most life-threatening complications [15].

Abnormal liver function tests and significantly increased aminotransferases are also associated with severe rhabdomyolysis. They can be caused by liver damage and muscle injury, as they are known to be released by both [16,17].

The creatine content in the supplement taken by our patient was listed as 3000 mg per dose plus caffeine and multiple other botanical ingredients. In a single serving, a 3000 mg “foundational creatine matrix” is made of a mixture of creatine monohydrate and creatine malate. This mixture has been well studied and is known to increase the phosphocreatine-to-creatine ratio in the skeletal muscle tissue. This change leads to rapid ATP re-synthesis during intensive exercise [18]. Moreover, with the use of creatine monophosphate and creatine malate, there is significant muscle swelling, and it is considered a key component for cell growth [14]. Performance enhancer users frequently take much higher doses of a supplement than recommended; Juhn et al. showed that 80% of athletes take a higher dose of creatine than recommended [15,19].

The supplement also had a 2000 mg of “nitric oxide blast complex” composed of arginine alpha-ketoglutarate, citrulline malate, L-arginine HCL, beta-alanine, and L-norvaline. These ingredients are supposed to increase nitric oxide levels to increase blood flow during exercise [20]. While L-arginine is a crucial amino acid required for synthesizing nitric oxide, studies suggest limited efficacy in improving blood flow and enhancing exercise performance [21]. Citrulline malate is another component of this exercise supplement; it has been shown to increase performance in individuals through its vasodilation properties. However, it is only productive if consumed in 6-8 grams daily doses [22].

Rhabdomyolysis is commonly associated with trauma and is defined as having a serum CK level greater than five times the upper limit of normal and a urine dipstick presenting myoglobinuria without hematuria [23,24]. Performance enhancers can lead to strenuous exercise, eventually causing rhabdomyolysis [25]. In a study among military recruits, approximately 40% displayed symptoms of rhabdomyolysis within six days of conditioning [26].

A potential complication of traumatic rhabdomyolysis is compartment syndrome, which is very rare in non-traumatic cases like those induced by exercise [27]. It is suggested that the edema from muscle and tissue damage and delineating fascia causes expansion and increased intercompartmental pressures, with lower extremities being affected more commonly [28,29]. Rhabdomyolysis-associated foot drop, like in our patient, is a rare occurrence. The proposed mechanism of peripheral neuropathy in rhabdomyolysis involves increased intramuscular pressure, creating a compression of the peroneal nerve, leading to foot drop and muscle damage, causing local inflammation, which may cause ischemia of the peroneal nerve [30-32].

The risk of peripheral neuropathy is correlated with increased serum CK levels, abnormal muscle intensity on imaging, and dense uptake on bone scans. Peripheral neuropathy is evaluated using nerve conduction studies and EMG [33,34].

These outcomes have been observed following moderate exercise and sometimes alcohol intake [35,36]. It's important to highlight that acute renal failure in these contexts has been induced not just by strenuous exercise alone but by a combination of factors, including supplement intake and lifestyle choices. It is not clear if the history of alcohol use may have contributed to his clinical condition.

Managing rhabdomyolysis requires evaluating and removing the potential causes, as well as immediate and aggressive hydration and correction of electrolyte and acid-base abnormalities [35]. In selected cases, preventing additional complications using sodium bicarbonate can help prevent myoglobin breakdown into the nephrotoxic metabolite called ferrihemate [36-38]. The overall prognosis of rhabdomyolysis is excellent when treated early, and most of the individuals have complete recovery of renal function, with approximately 4% requiring hemodialysis [39-41].

In summary, our patient presented with severe rhabdomyolysis, life-threatening electrolyte and acid-base imbalance, dehydration with hemoconcentration, renal and liver injury, and peripheral neuropathy. This was associated with the use of performance-enhancing supplements and exercise. He required hemodialysis in addition to aggressive management to stabilize his condition. He had residual peripheral neuropathy damage on discharge, and his long-term recovery is uncertain. Fortunately, he did not require long-term hemodialysis [42]. 

## Conclusions

This case study highlights the inherent risks and medical complications stemming from using unregulated exercise aids and supplements. Despite the widespread marketing of these products as "natural" and "safe," our findings point to a troubling increase in patients suffering from adverse effects linked to their use. These insights demonstrate the potential risks associated with the use of pre-workout supplements and the importance of being vigilant about the signs and symptoms of rhabdomyolysis, compartment syndrome, and renal failure. Healthcare professionals across various specialties must be vigilant in screening for and addressing supplement-associated complications, emphasizing the importance of gathering comprehensive information about all non-prescribed substances their patients may be consuming. This collective awareness and proactive approach are vital in mitigating the potential health risks posed by these unregulated products.
